# Membrane current evoked by mitochondrial Na^+^–Ca^2+^ exchange in mouse heart

**DOI:** 10.1186/s12576-020-00752-3

**Published:** 2020-04-30

**Authors:** Mohammed M. Islam, Ayako Takeuchi, Satoshi Matsuoka

**Affiliations:** 1 Department of Integrative and Systems Physiology, Faculty of Medical Sciences, University of Fukui, 23-3 Matsuokashimoaizuki, Eiheiji-cho, Yoshida-gun, Fukui, 910-1193 Japan; 2 Life Science Innovation Center, University of Fukui, Fukui, 910-1193 Japan

**Keywords:** Mitochondria, Electrophysiology, Na^+^–Ca^2+^ exchange, Heart

## Abstract

The electrogenicity of mitochondrial Na^+^–Ca^2+^ exchange (NCXm) had been controversial and no membrane current through it had been reported. We succeeded for the first time in recording NCXm-mediated currents using mitoplasts derived from mouse ventricle. Under conditions that K^+^, Cl^−^, and Ca^2+^ uniporter currents were inhibited, extra-mitochondrial Na^+^ induced inward currents with 1 μM Ca^2+^ in the pipette. The half-maximum concentration of Na^+^ was 35.6 mM. The inward current was diminished without Ca^2+^ in the pipette, and was augmented with 10 μM Ca^2+^. The Na^+^-induced inward currents were largely inhibited by CGP-37157, an NCXm blocker. However, the reverse mode of NCXm, which should be detected as an outward current, was hardly induced by extra-mitochondrial application of Ca^2+^ with Na^+^ in the pipette. It was concluded that NCXm is electrogenic. This property may be advantageous for facilitating Ca^2+^ extrusion from mitochondria, which has large negative membrane potential.

## Background

Mitochondrial Ca^2+^ has pivotal roles in mitochondrial metabolism, apoptosis, and cytoplasmic Ca^2+^ signaling [1–5]. Mitochondrial Ca^2+^ influx is mainly mediated via mitochondrial Ca^2+^ uniporter (MCU), and efflux via Na^+^–Ca^2+^ exchanger (NCXm) and H^+^–Ca^2+^ exchanger. MCU is a Ca^2+^ channel, whose flux is driven by the negative mitochondrial membrane potential (ΔΨ), − 180 mV, as demonstrated in the patch clamp experiments using mitoplasts [6]. H^+^–Ca^2+^ exchanger is likely to be electroneutral, exchanging two H^+^ with one Ca^2+^ [1]. However, the electrophysiological property of NCXm has been unknown.

Carafoli et al. first discovered NCXm [7], namely Na^+^-dependent mitochondrial Ca^2+^ efflux, in rat heart, and Palty et al. identified NCLX as an essential component of NCXm [8]. Since the study by Palty et al. [8], physiological significances of NCLX or NCXm have been extensively elucidated in many types of cell, such as automaticity of cultured cardiomyocytes [9], insulin secretion of pancreatic β cells [10, 11], control of nociceptive signaling in dorsal root ganglion neurons [12], B cell receptor-mediated Ca^2+^ signaling and chemotaxis of B lymphocytes [13–15], and Ca^2+^ oscillation in depolarized mitochondria of mast cells [16]. The tamoxifen-induced knockout of NCLX in mouse heart resulted in severe myocardial dysfunction and heart failure [17]. In addition, it was reported that NCLX is associated with a familial form of Parkinson’s disease where PINK-1 deficiency leads to impairment of mitochondrial Ca^2+^ efflux [18] and also with a progression of Alzheimer’s disease [19]. Despite the importance of NCXm in physiological and pathophysiological cell functions, its dependence on ΔΨ, which is the key biophysical property for mitochondrial ATP synthesis, has been controversial.

Early studies using fluorescence probes and Ca^2+^-sensitive electrode suggested that NCXm activity depends on ΔΨ, based on findings of a high Hill coefficient for cytoplasmic Na^+^ (∼ 3) and the attenuation of Na^+^-dependent Ca^2+^ efflux by mitochondrial depolarization in rat heart mitochondria [20, 21]. Jung et al. later supported the ΔΨ-dependence of NCXm by showing the disruption of Ca^2+^ efflux through NCXm by dissipation of ΔΨ in beef heart mitochondria [22]. To the contrary, other studies suggested electroneutral exchange via NCXm. Affolter and Carafoli demonstrated that ΔΨ did not alter when the Ca^2+^ efflux via NCXm was induced using rat heart mitochondria [23]. Brand [24] and Wingrove and Gunter [25] supported this idea by showing a Hill coefficient of two for Na^+^ using rat heart and liver mitochondria, respectively. Later, using imaging methods, we demonstrated the reversal of NCXm activity by ΔΨ depolarization and NCXm activity-dependent change of ΔΨ in permeabilized rat cardiomyocytes, and suggested that NCXm is voltage dependent and electrogenic [26]. Dash and Beard also supported the electrogenic property with a stoichiometry of 3:1 by comparing simulation results of a mathematical model of mitochondria with NCXm of 3:1 or 2:1 stoichiometry to the experimental data [27]. The controversy has been caused by difficulty in direct measurement of membrane current through NCXm. Here we succeeded, for the first time, in recording and characterizing the NCXm-mediated current using mitoplasts derived from mouse heart.

## Methods

### Animals

C57BL/6J mice were housed in a 12 h light–dark cycle with ad libitum access to food and water. The experimental protocols were approved by Animal Research Committee, University of Fukui.

### Isolation of mouse ventricular mitochondria by differential centrifugation

10–16 week old C57BL/6J mice were heparinized (200 U/mouse, i.p.) and sacrificed by cervical dislocation. The ventricular mitochondria were isolated by a conventional differential centrifugation method. The heart was quickly excised after thoracotomy and placed on an ice-cold sucrose buffer with 0.05% bovine serum albumin (BSA), and the atria and other debris were removed to isolate the ventricular tissues. The ventricular tissue was then cut into pieces and homogenized in the sucrose buffer with 0.05% BSA. The homogenate was centrifuged for 10 min at 8500*g*. The pellet was re-suspended in the sucrose buffer with 0.05% BSA, and centrifuged for 10 min at 800*g*. The resulting pellet contains nucleus and unbroken cells. To collect the mitochondria, the supernatant was centrifuged for 10 min at 8500*g*, washed with the sucrose buffer without BSA, and the resulting pellet which contains mitochondria was re-suspended in 100 μl sucrose buffer without BSA and stored on ice. The sucrose buffer contained 250 mM sucrose, 5 mM HEPES, and 1 mM EGTA (pH 7.2 with KOH). All centrifugation steps were performed at 2 °C.

### Western blot analysis

Isolated mitochondria (25 μg) were lysed with a Laemmli sample buffer (Bio-Rad) containing β-mercaptoethanol, denatured at 55 °C for 30 min, resolved by SDS-PAGE and transferred to PVDF membranes (Bio-Rad). Blots were incubated for 1 h at room temperature in blocking one (Nacalai tesque), then incubated in primary antibodies, anti-MCU (Cell Signaling Technology; 1:1000) or anti-COX IV (abcam; 1:2000) for 1 h. Blots were incubated with HRP-linked 2nd antibodies for 30 min. The images were developed with ECL Prime Western Blotting Detection Reagent (GE Healthcare) and acquired by ChemiDoc XRS Plus (Bio-Rad).

### Mitoplast preparation

Isolated mitochondria were centrifuged for 10 min at 8500*g*. The pellet was given hypotonic shock for 5 min to yield mitoplasts. The mitoplasts were collected by centrifugation for 5 min at 8500*g*. The pellet was suspended in a storage buffer. The entire procedure was performed on ice and ice-cold solutions were used. The mitoplast suspension was stored on ice and used within 4–5 h. The hypotonic solution contained 5 mM sucrose, 5 mM HEPES, and 1 mM EGTA (pH 7.2 with KOH), and the storage buffer contained 750 mM KCl, 100 mM HEPES, and 1 mM EGTA (pH 7.2 with KOH).

### Labeling of mitochondria and confocal imaging

The isolated mitochondria were loaded with 2 μM MitoTracker Green FM (ThermoFisher Scientific) for 1 h on ice. The confocal images were taken using a confocal microscope with a ×100 objective lens with the excitation at 473 nm and the emission at 485–585 nm (Olympus FV1200).

### Ca^2+^ uptake assay

For measurement of extra-mitochondrial Ca^2+^, isolated mitochondria (75 μg) were suspended in an assay buffer (100 μl) containing 125 mM KCl, 2 mM K_2_HPO_4_, 20 mM HEPES, 0.01 mM EGTA, 2 mM MgCl_2_, 1 mM malate, 7 mM potassium pyruvate, 5 μM cyclosporin A (Sigma-Aldrich), and 0.5 μM Calcium Green-5N (pH 7.2 with KOH), then were placed in 96-well plate. The extra-mitochondrial Ca^2+^ was evaluated by measuring fluorescence using a multimode plate reader (Enspire, PerkinElmer), with the excitation at 505 nm and the emission at 530 nm. At 410 s, mitochondria were challenged by 50 μM CaCl_2_ in the presence or absence of 25 mM NaCl, to initiate Ca^2+^ influx into mitochondria via MCU. At 1000 s, an MCU blocker, 5 μM Ru360 (Calbiochem), was added to facilitate Ca^2+^ efflux from mitochondria. The initial Ca^2+^ efflux velocity was calculated by fitting linearly to the data for initial 60 s after the addition of Ru360.

For measurement of intra-mitochondrial Ca^2+^, isolated mitochondria (50 μg) were loaded with 20 μM Fluo-8, AM (AAT Bioquest) for 30 min at 25 °C, followed by washing twice. The resulting mitochondria in assay buffer (200 μl) containing 125 mM KCl, 2 mM K_2_HPO_4_, 20 mM HEPES, 0.01 mM EGTA, 2 mM MgCl_2_, 1 mM malate, 7 mM potassium pyruvate, and 5 μM cyclosporin A (pH 7.2 with KOH) were placed in 96-well plate. The intra-mitochondrial Ca^2+^ was evaluated by measuring fluorescence with the excitation at 490 nm and the emission at 514 nm. At 300 s, mitochondria were challenged by 20 μM CaCl_2_ in the presence or absence of 5 μM Ru360 or 10 μM ruthenium red (Wako), to initiate Ca^2+^ influx into mitochondria via known Ca^2+^ influx systems including MCU. The initial Ca^2+^ influx velocity was calculated by fitting linearly to the data for initial 26 s after the addition of 20 μM CaCl_2_.

To induce exchange of intra-mitochondrial Na^+^ with extra-mitochondrial Ca^2+^, i.e., reverse mode of NCXm, the Fluo-8, AM loading procedure was performed in the presence of a Na^+^ ionophore 4 μM monensin and of a Na^+^–H^+^ antiporter blocker 100 μM ethylisopropyl amiloride (EIPA; Tocris Biosciences) in Mg^2+^-free assay buffer where 125 mM KCl was replaced with 125 mM NaCl to facilitate mitochondrial Na^+^ accumulation. The resulting mitochondria were re-suspended in assay buffer (200 μl) containing 125 mM KCl, 25 mM Na^+^, 2 mM K_2_HPO_4_, 20 mM HEPES, 0.01 mM EGTA, 2 mM MgCl_2_, 1 mM malate, 7 mM potassium pyruvate, 100 μM EIPA, 10 μM ruthenium red, and 5 μM cyclosporin A (pH 7.2 with KOH). Since mitochondrial depolarization facilitated reverse mode of NCXm [26], 10 μM antimycin A (Sigma-Aldrich) and 2 μM oligomycin (Sigma-Aldrich) were also added. To evaluate intra-mitochondrial Na^+^-induced extra-mitochondrial Ca^2+^ influx, the same protocol was performed in the absence of Na^+^ throughout the experiment for comparison. The initial Ca^2+^ influx velocity was calculated by fitting linearly to the data for initial 26 s after the addition of 20 μM CaCl_2_.

### Electrophysiology

A perfusion chamber was equipped on an inverted microscopy (TE2000-U, Nikon) with a ×100 objective lens. The glass bottom of chamber was pre-treated for 5 min with a KCl-divalent free (KCl-DVF) solution containing 0.05% BSA, to prevent adhesion of mitoplasts. The mitoplast suspension of 3–5 μl was added to 35 μl of the KCl-DVF solution on the bottom of chamber. Solitary mitoplasts with a diameter of ~ 2–3 μm and with round shape were selected for whole-mitoplast patch. The mitoplasts were initially perfused with the KCl-DVF solution. Bath solution was changed within 5 s using a perfusion controller (Valvelink-8.2, Automate scientific). The KCl-DVF solution contained 150 mM KCl, 10 mM HEPES, and 1 mM EGTA (pH 7.2 with KOH).

Voltage clamp experiment was performed using a patch clamp amplifier (Axopatch 200B, Molecular Devices), a digitizer (Digidata 1440A, Molecular Devices) and a software (pClamp 10.7, Molecular Devices). All data were sampled at 10 kHz and later filtered at 2 kHz. For graphical presentation, the membrane current traces were further filtered at 500 Hz (Gaussian filter). Pipettes were prepared using a puller (PC-100, Narishige). After the formation of GΩ seal, the capacitance was compensated and voltage pulses of 350–700 mV for 5–10 ms duration were applied to rupture the membrane and to form the whole mitoplast configuration. The capacitance of mitoplast was 0.1–1.0 pF (0.32 ± 0.22 pF, *n* = 34). The membrane potential was held at 0 mV, and the ramp pulse of 900 ms duration from −160 to 80 mV was applied every 10 s.

To measure the Na^+^-induced inward NCXm current, a Na^+^-free and Ca^2+^-containing pipette solution was used, and the bath solution was changed from a Na^+^-free and Ca^2+^-free bath solution to a Na^+^-containing bath solution. The Na^+^-free and Ca^2+^-containing pipette solution contained 30 mM tetramethylammonium hydroxide (TMA-OH), 2 mM HCl, 100 mM HEPES, and 1.5 mM EGTA. 0, 1.038 and 1.5 mM CaCl_2_ were added to get 0, 1 and 10 μM free Ca^2+^, respectively (pH 7.2 with d-gluconic acid). The free Ca^2+^ concentration was calculated with an online calculator, WEBMAXC standard (https://somapp.ucdmc.ucdavis.edu/pharmacology/bers/maxchelator/webmaxc/webmaxcS.htm). The pipette resistance was 30–40 MΩ when filled with the pipette solution. The Na^+^-free and Ca^2+^-free bath solution contained 145 mM HEPES, 50 mM Tris, and 5 mM EGTA (pH 7.2 with Tris). The Na^+^-containing bath solution contained 145 mM HEPES, 50 mM NaOH, and 5 mM EGTA (pH 7.2 with Tris).

To measure the Ca^2+^-induced outward Na^+^–Ca^2+^ exchange current, a Na^+^-containing pipette solution was used; 30 mM TMA-OH, 100 mM NaOH, 2 mM HCl, 100 mM HEPES, 1.5 mM EGTA, and 0.6058 mM CaCl_2_ (pH 7.5 with d-gluconic acid). Calculated free Ca^2+^ concentration was 0.1 μM. A Ca^2+^-free bath solution contained 145 mM HEPES, 30 mM Tris, 20 mM NaOH, 5 mM EGTA, and 3.2 mM HCl (pH 7.2 with HEPES). Composition of 1 mM Ca^2+^-containing bath solution was 145 mM HEPES, 30 mM Tris, 20 mM NaOH, 1.2 mM HCl, 0 mM EGTA, and 1 mM CaCl_2_ (pH 7.2 with HEPES).

To block the mitochondrial Ca^2+^ uniporter, 1 μM Ru360 was added to all the bath solutions (IC50 of 184 pM [28]). To block the NCXm, 2 μM CGP-37157 (Tocris Bioscience) was added to the bath solutions (IC50 of 0.36 μM [29]).

An average of three consecutive membrane currents in response to ramp pulses was used for analysis in each condition. All electrophysiological recordings were performed under continuous perfusion of bath solutions at 22–25 °C.

### Statistical analysis

All data are demonstrated as mean ± s.e.m. of individual experiments which are presented as *n*. Statistical analyses were performed with unpaired two-tailed Student’s *t* test for two-group comparisons or with one-way ANOVA multiple comparisons, followed by Student–Newman–Keuls Method, respectively, using SigmaPlot 14 (Systat Software, Inc.). *p* < 0.05 was considered significant.

## Results

### Quality assessment of mitochondria

**Fig. 1:**
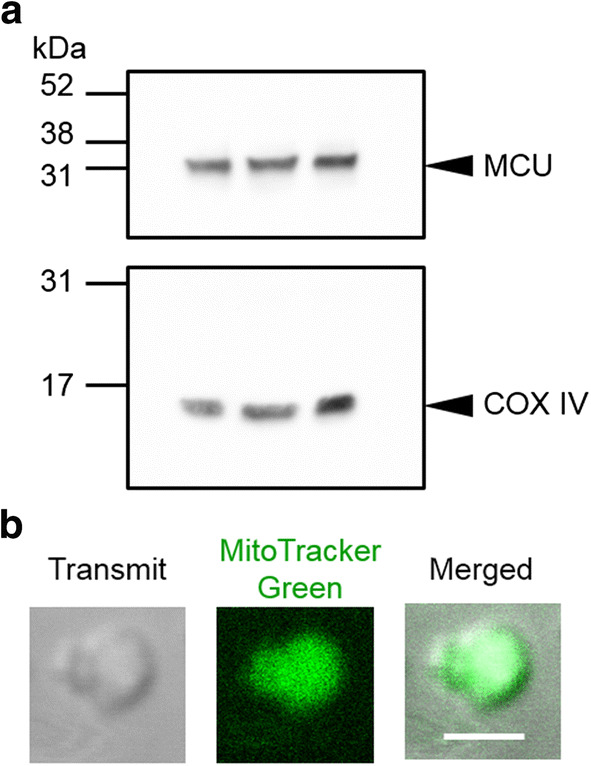
Quality assessment of mitochondria. **a** Western blot analyses of mitochondria isolated from mouse ventricle. Mitochondrial identity was confirmed by assessing the expressions of MCU and COX IV. **b** A mitoplast stained with MitoTracker Green. Transmit, fluorescent and merged images of a mitoplast are shown. A bar indicates 2 μm

We first examined the quality of the isolated mitochondria. Western blot analysis confirmed the expression of mitochondrial proteins, MCU and COX IV, in the preparations (Fig. 1a). The quality of mitoplast was evaluated using MitoTracker Green-loaded mitochondria (Fig. 1b). The transmit image of a mitoplast clearly showed the inner mitochondrial membrane as a circle and the remnants of ruptured outer mitochondrial membrane. The fluorescent and merged images of mitoplasts confirmed that the mitoplast originated from mitochondria.

**Fig. 2:**
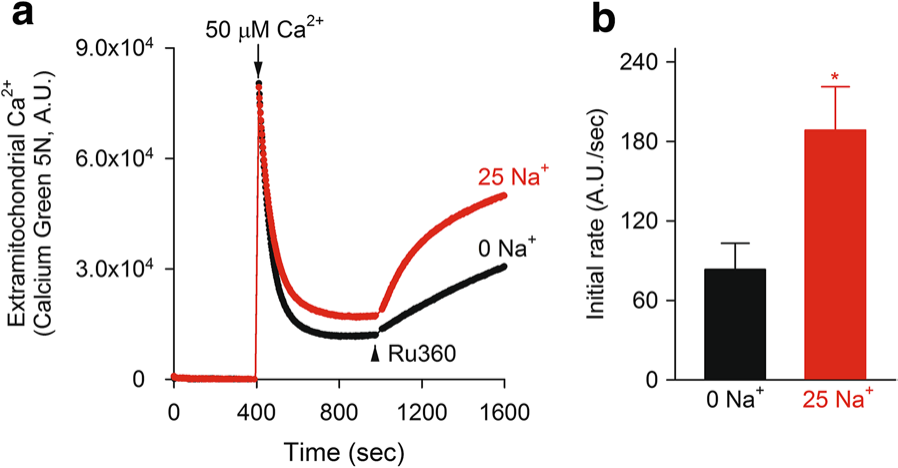
Na^+^-induced Ca^2+^ efflux from isolated mitochondria. **a** Representative traces of mitochondrial Ca^2+^ uptake assay. Blocking Ca^2+^ influx through MCU by 5 μM Ru360 induced Ca^2+^ efflux from mitochondria, which was accelerated by extra-mitochondrial Na^+^ (25 mM). **b** Initial Ca^2+^ efflux rate in the absence and presence of extra-mitochondrial Na^+^ (*n* = 4). *p < 0.05

Mitochondrial Ca^2+^ uptake and extrusion were evaluated by monitoring extra-mitochondrial Ca^2+^ with Calcium Green 5N (Fig. 2a). An addition of 50 μM Ca^2+^ caused a transient rise and subsequent decline of extra-mitochondrial Ca^2+^, suggesting Ca^2+^ uptake by MCU. The following addition of Ru360, an inhibiter of MCU, induced Ca^2+^ efflux, which had two components, extra-mitochondrial Na^+^-dependent and -independent. The Na^+^-dependent Ca^2+^ efflux was defined as NCXm and its Ca^2+^ transporting rate was about 1.5-fold larger than that of Na^+^-independent Ca^2+^ efflux (Fig. 2b). The results confirm that NCXm does exist in mouse ventricular mitochondria.

### Na^+^-induced inward current

**Fig. 3:**
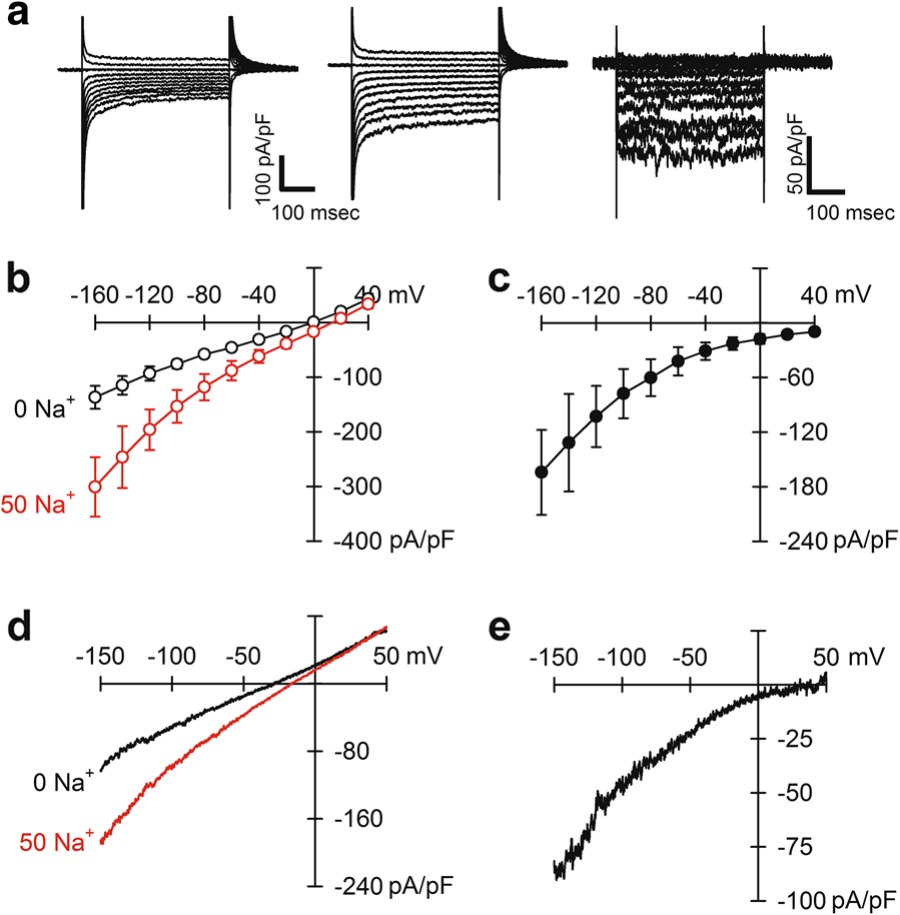
Na^+^-induced inward current. **a** Membrane currents in the absence (left) and presence (middle) of 50 mM Na^+^ in the bath solution. The difference currents, middle minus left, are shown in the right. **b** Current–voltage relationships in the absence (black) and presence (red) of 50 mM Na^+^ in the bath solution (*n* = 4). The amplitude was measured near the end of square pulses. **c** The difference currents derived from **b** (*n* = 4). Square pulse protocol was used in **a–c**. **d** Representative current–voltage relationships in the absence (black) and presence (red) of 50 mM Na^+^ in the bath solution. **e** The difference current derived from **d**. Ramp voltage pulse protocol was used in **d** and **e**. The pipette solution contained 1 μM free Ca^2+^ in all experiments

The membrane currents were first measured using square pulses with a holding potential of 0 mV changing to − 160 ∼ + 40 mV every 20 mV with no Na^+^ and 1 μM Ca^2+^ in the pipette solution. An application of extra-mitochondrial 50 mM Na^+^ augmented the amplitude of inward currents (a middle panel vs a left panel of Fig. 3a). The difference currents between those in the presence and absence of Na^+^ demonstrated Na^+^ induced-inward currents, which are time-independent (a right panel of Fig. 3a). The summarized current–voltage relations are shown in Fig. 3b, c, demonstrating Na^+^-induced inward currents at all membrane potentials examined (Fig. 3c). Since the Na^+^-induced current was time-independent, we employed a ramp voltage protocol of 900 ms duration. The ramp voltage protocol demonstrated Na^+^-induced inward currents essentially similar to those with the square pulse protocol (Fig. 3d, e). In the following experiments, we employed the ramp voltage pulse protocol.

### Inhibition of Na^+^-induced inward current by CGP-37157

**Fig. 4:**
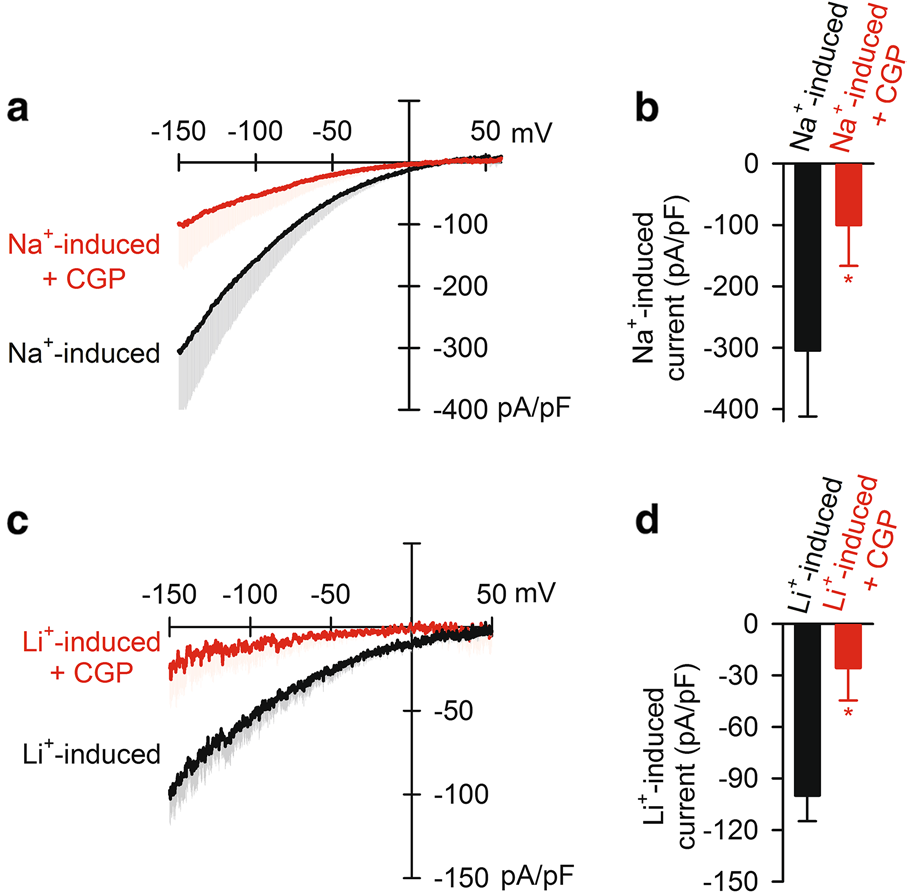
Inhibition of Na^+^- or Li^+^-induced inward current by CGP-37157. **a** Current–voltage relationships of 50 mM Na^+^-induced current in the absence (black) and presence (red) of 2 μM CGP-37157 (*n* = 4). **b** The current amplitude at −150 mV. **c** Current–voltage relationships of 50 mM Li^+^-induced current in the absence (black) and presence (red) of 2 μM CGP-37157 (*n* = 4). **d** The current amplitude at −150 mV. In all experiments, pipette solution contained 1 μM free Ca^2+^. *p < 0.05. CGP: CGP-37157

Next, we examined the effects of CGP-37157, a well-known NCXm blocker, on the Na^+^-induced inward current. 2 μM CGP-37157 significantly blocked the Na^+^-induced inward current by 67% (Fig. 4a, b), suggesting the current was mediated by NCXm, at least in part.

It has been recognized that NCXm is able to operate exchange of Li^+^ with Ca^2+^ [8, 20], unlike the plasma membrane Na^+^–Ca^2+^ exchange. Consistent with the unique property, 50 mM Li^+^ induced inward currents which were comparable to the Na^+^-induced inward current with a lower magnitude (Fig. 4c, d). CGP-37157 blocked the Li^+^-induced current by 74%, similarly to the Na^+^-induced current (Fig. 4b, d). These data suggested that the Na^+^- or Li^+^-induced inward current was mediated by NCXm.

### Na^+^ and Ca^2+^ concentration dependences of Na^+^-induced current

**Fig. 5:**
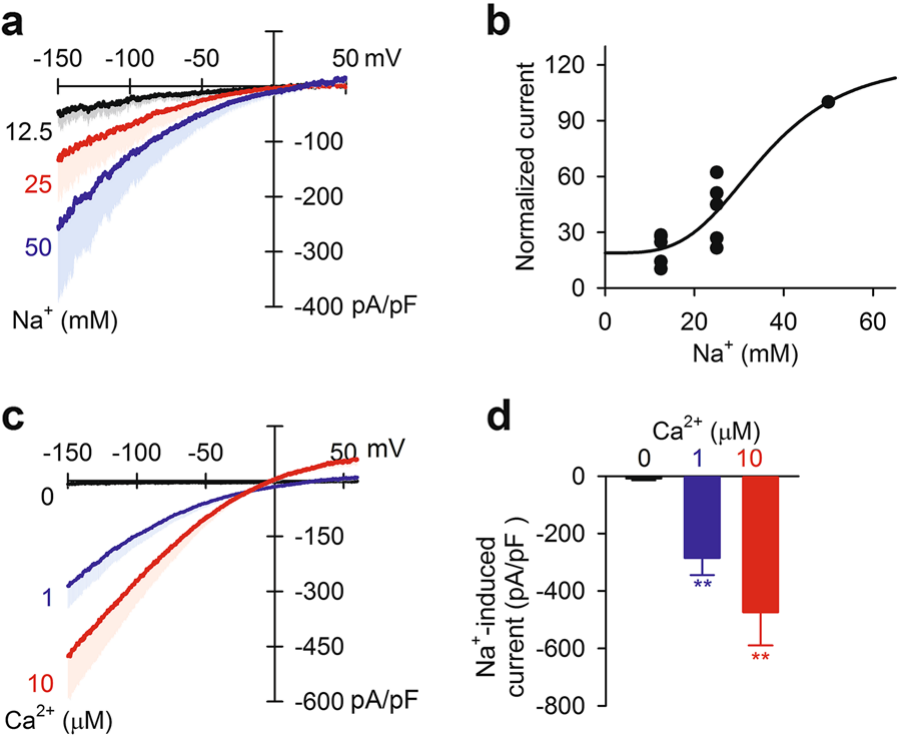
Na^+^- and Ca^2+^-concentration dependences of Na^+^-induced inward current. **a** Current–voltage relationships of Na^+^-induced current with extra-mitochondrial 12.5, 25 and 50 mM Na^+^ (*n* = 5). **b** Extra-mitochondrial Na^+^ concentration dependence of Na^+^-induced inward current. Current was measured at −150 mV and normalized to the current induced by 50 mM Na^+^. Fitted half-maximum concentration and Hill coefficient are 35.6 mM and 3.7, respectively. **c** Current–voltage relationships of 50 mM Na^+^-induced current with 0, 1 and 10 μM Ca^2+^ in the pipette solution (*n* = 6–15). **d** Intra-mitochondrial Ca^2+^ concentration dependence of inward current at −150 mV. **p < 0.01 vs current without Ca^2+^ in pipette

Extra-mitochondrial Na^+^ dependence was examined in Fig. 5a, b. The current was clearly dependent on extra-mitochondrial Na^+^ concentration, with a half-maximum concentration of 35.6 mM. We further studied intra-mitochondrial Ca^2+^ dependence by altering the Ca^2+^ concentration in the pipette solution. Without Ca^2+^ in the pipette solution, the application of 50 mM Na^+^ hardly induced inward current. Conversely, with 10 μM Ca^2+^ in the pipette solution, the Na^+^-induced current markedly increased (Fig. 5c, d).

### Ca^2+^-induced outward current

**Fig. 6:**
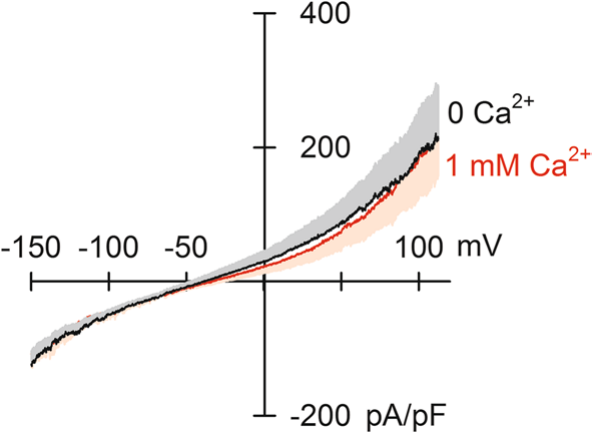
Extra-mitochondrial Ca^2+^-induced current. Current–voltage relationships in the absence and presence of 1 mM Ca^2+^ in the bath solution (*n* = 4). 1 mM extra-mitochondrial Ca^2+^ with 100 mM Na^+^ in the matrix side did not induce outward current

It was reported that NCXm could operate in a reverse mode, i.e. Ca^2+^ uptake in exchanging with mitochondrial Na^+^ [16, 22, 26, 30, 31]. We tried to detect outward current by applying extra-mitochondrial Ca^2+^ with high-concentration Na^+^ in the pipette (100 mM). Contrary to our expectation, extra-mitochondrial Ca^2+^, up to 1 mM, hardly evoked outward current (Fig. 6).

**Fig. 7:**
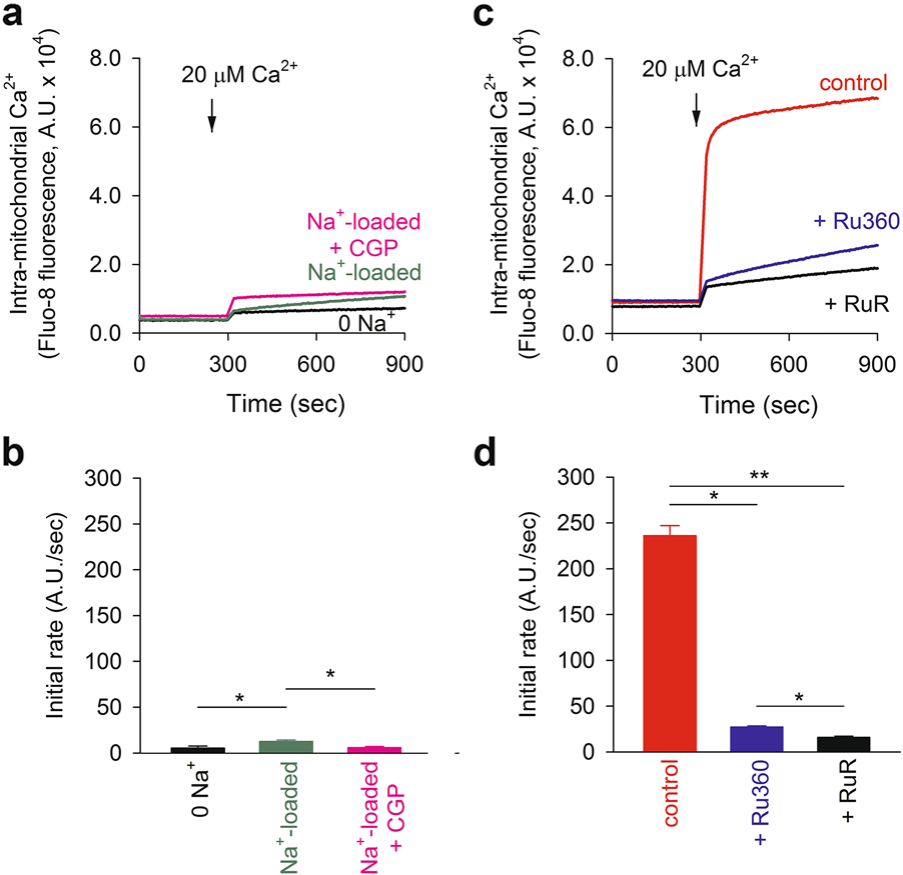
Ca^2+^ uptake through reverse mode of NCXm in isolated mitochondria. **a**, **b** Ca^2+^ uptake in the depolarized mitochondria. Intra-mitochondrial Ca^2+^ was measured in Na^+^-unloaded mitochondria (0 Na^+^), Na^+^-loaded mitochondria (Na^+^-loaded) and Na^+^-loaded mitochondria with 20 μM CGP-37157 (Na^+^-loaded + CGP). **a** Representative data. **b** Initial rates of intra-mitochondrial Ca^2+^ rise (*n* = 4). *p < 0.05. **c**, **d** Ca^2+^ uptake in energized mitochondria. Intra-mitochondrial Ca^2+^ uptake was measured in control (control), in the presence of Ru360 (+ Ru360) and in the presence of ruthenium red (+ RuR). **c** Representative data. **d** Initial rates of intra-mitochondrial Ca^2+^ rise (*n* = 4). *p < 0.05

To explore reasons why the outward NCXm current was not detectable, we evaluated Ca^2+^ uptake via the reverse mode of NCXm by measuring intra-mitochondrial Ca^2+^ using Fluo-8. In the mitochondria loaded with Na^+^, an application of 20 μM Ca^2+^ under the condition of mitochondrial depolarization induced a slow increase in intra-mitochondrial Ca^2+^, whose rate was only 1.6 times faster than that of Na^+^-unloaded mitochondria (Fig. 7a, b). The slow Ca^2+^ rise was blocked by 2 μM CGP-37157, suggesting that the Ca^2+^ rise was mediated via the reverse mode of NCXm (Fig. 7a, b). Contrarily, the rate of Ca^2+^ uptake though known Ca^2+^ influx systems including MCU under the condition of normal ΔΨ was larger by an order of magnitude than that of the reverse mode of NCXm; Ru360 and ruthenium red-sensitive Ca^2+^ uptake rate was 208.7 ± 11.8 A.U./s and 219.9 ± 11.6, respectively (Fig. 7c, d), and CGP-37157-sensitive Na^+^-dependent Ca^2+^ uptake rate was 6.7 ± 0.4 A.U./s (*n* = 4) (Fig. 7a, b). The slower turnover of reverse mode of NCXm may hamper recording of membrane current mediated by the reverse mode of NCXm.

## Discussion

In this study, we report, for the first time, the membrane current mediated by NCXm using the whole-mitoplast patch clamp technique. The recorded inward current is most likely mediated by the NCXm based on the Na^+^ and Li^+^ selectively, the requirement of Ca^2+^ in the opposite membrane site, and the inhibition by CGP-37157. This study provides a final conclusion on the electrogenicity of NCXm which has been controversial for long time [20–27].

A variety of half-maximum concentration values of NCXm for extra-mitochondrial Na^+^ has been reported in cardiac myocytes and other cells, ranging from 0.9 to 12 mM [11, 20, 25, 26]. The half-maximum concentration of 35.6 mM, derived from the present study, is rather high. This might be partly because contamination of Na^+^-permeable current could not be completely eliminated under our conditions. Further detailed examinations for clarifying the origin of Na^+^-leak currents are necessary to solve the problem. Nevertheless, the cytosolic Na^+^ dependence of NCXm may have an important role under the conditions of heart failure, in which situation both cytosolic Ca^2+^ and Na^+^ tend to increase [32–34]. The rise of cytosolic Ca^2+^ may induce mitochondrial Ca^2+^ overload and dysfunction. However, the elevation of cytosolic Na^+^ may facilitate mitochondrial Ca^2+^ efflux through NCXm and protect mitochondrial dysfunction [35].

The stoichiometry of NCXm is an important issue. The fact that the exchange of extra-mitochondrial Na^+^ with intra-mitochondrial Ca^2+^ produces inward current indicates the stoichiometry 3 or more Na^+^:1 Ca^2+^ exchange. However, it was not possible to derive it from reversal potentials because of the relatively small current density and the difficulty in inducing the outward current. The reverse mode of NCXm was described earlier [16, 22, 26, 30, 31]. However, the mitochondrial Ca^2+^ rise through the reverse mode of NCXm was relatively slow and it took more than several tens of minutes to reach a steady state [26]. Consistent with the previous observation, the intra-mitochondrial Ca^2+^ rise though reverse mode of NCXm was much slower than that of MCU (Fig. 7). It should be noted that the rate of extra-mitochondrial Ca^2+^ change through the forward mode of NCXm is in a level comparable to that of MCU (Fig. 2). Therefore, it may be reasonable to speculate that the operation of reverse mode of NCXm is much slower than the forward mode. NCXm might go into an inactivated state at higher intra-mitochondrial Na^+^ concentrations, in a similar manner to plasma membrane NCX [36]. A method for faster ion concentration jump, which was used for NCX current study, may be useful for studying the possible inactivation, but it could not be used in this study because of relatively fragile nature of mitoplast. Further study is needed to clarify the mechanisms of slower operation of reverse mode and to determine the stoichiometry.

In this study, we used CGP-37157 at a sub-saturating concentration, 2 μM, in the electrophysiological experiments to avoid its possible off-target effects. It has been reported that CGP-37157 affects many ion channels and transporters including plasma membrane Na^+^–Ca^2+^ exchange at higher concentrations [37–39]. Palty et al. reported that 5 μM CGP-37157 exhibited approximately 50% inhibition of Ca^2+^ efflux from mitochondria [8]. The effect of 2 μM CGP-37157 on the Na^+^-induced current (67%) is comparable to the inhibitory effects shown in the previous report. Therefore, the CGP-37157-sensitive current component is most likely to be mediated by NCXm. However, we assume that other membrane currents such as leak current were, at least partially, contaminated when 10 μM Ca^2+^ was included in the pipette solution because outward current was induced (Fig. 5c).

ΔΨ is about −180 mV with reference to cytosol, and are the energy source for ATP synthesis from ADP and inorganic phosphate by ATP synthase. The ΔΨ strongly facilitates Ca^2+^ entering into mitochondria through MCU and other Ca^2+^-permeable systems. The voltage-dependent nature of NCXm is advantageous for Ca^2+^ extrusion over electroneutral exchange. Therefore, ΔΨ depolarization under the pathological conditions such as ischemia should attenuate both Ca^2+^ influx and efflux, resulting in severe damage of mitochondria.

## Conclusions

The mitochondrial Na^+^– or Li^+^–Ca^2+^ exchange current was identified for the first time in mitoplasts derived from mouse ventricle. It was concluded that NCXm is electrogenic with a stoichiometry of 3 or more Na^+^:1 Ca^2+^.

## Data Availability

All data generated or analyzed during this study are included in this published article.

## Abbreviations

NCXm: Mitochondrial Na^+^–Ca^2+^ exchange
MCU: Mitochondrial Ca^2+^ uniporter
ΔΨ: Mitochondrial membrane potential
BSA: Bovine serum albumin
EIPA: Ethylisopropyl amiloride
KCl-DVF: KCl-divalent free
TMA-OH: Tetramethylammonium hydroxide

## References

[1] Bernardi P (1999). Mitochondrial transport of cations: channels, exchangers, and permeability transition. Physiol Rev.

[2] Takeuchi A, Kim B, Matsuoka S (2015). The destiny of Ca^2+^ released by mitochondria. J Physiol Sci.

[3] Saito R, Takeuchi A, Himeno Y, Inagaki N, Matsuoka S (2016). A simulation study on the constancy of cardiac energy metabolites during workload transition. J Physiol.

[4] Kostic M, Sekler I (2019). Functional properties and mode of regulation of the mitochondrial Na^+^/Ca^2+^ exchanger, NCLX. Semin Cell Dev Biol.

[5] Takeuchi A, Matsuoka S (2020). Integration of mitochondrial energetics in heart with mathematical modelling. J Physiol.

[6] Kirichok Y, Krapivinsky G, Clapham DE (2004). The mitochondrial calcium uniporter is a highly selective ion channel. Nature.

[7] Carafoli E, Tiozzo R, Lugli G, Crovetti F, Kratzing C (1974). The release of calcium from heart mitochondria by sodium. J Mol Cell Cardiol.

[8] Palty R, Silverman WF, Hershfinkel M, Caporale T, Sensi SL, Parnis J, Nolte C, Fishman D, Shoshan-Barmatz V, Herrmann S, Khananshvili D, Sekler I (2010). NCLX is an essential component of mitochondrial Na^+^/Ca^2+^ exchange. Proc Natl Acad Sci U S A.

[9] Takeuchi A, Kim B, Matsuoka S (2013). The mitochondrial Na^+^–Ca^2+^ exchanger, NCLX, regulates automaticity of HL-1 cardiomyocytes. Sci Rep.

[10] Nita II, Hershfinkel M, Fishman D, Ozeri E, Rutter GA, Sensi SL, Khananshvili D, Lewis EC, Sekler I (2012). The mitochondrial Na^+^/Ca^2+^ exchanger upregulates glucose dependent Ca^2+^ signalling linked to insulin secretion. PLoS ONE.

[11] Nita II, Hershfinkel M, Kantor C, Rutter GA, Lewis EC, Sekler I (2014). Pancreatic beta-cell Na^+^ channels control global Ca^2+^ signaling and oxidative metabolism by inducing Na^+^ and Ca^2+^ responses that are propagated into mitochondria. FASEB J.

[12] Nita II, Caspi Y, Gudes S, Fishman D, Lev S, Hersfinkel M, Sekler I, Binshtok AM (2016). Privileged crosstalk between TRPV1 channels and mitochondrial calcium shuttling machinery controls nociception. Biochim Biophys Acta.

[13] Kim B, Takeuchi A, Koga O, Hikida M, Matsuoka S (2012). Pivotal role of mitochondrial Na^+^–Ca^2+^ exchange in antigen receptor mediated Ca^2+^ signalling in DT40 and A20 B lymphocytes. J Physiol.

[14] Kim B, Takeuchi A, Hikida M, Matsuoka S (2016). Roles of the mitochondrial Na^+^–Ca^2+^ exchanger, NCLX, in B lymphocyte chemotaxis. Sci Rep.

[15] Takeuchi A, Kim B, Matsuoka S (2020). Physiological functions of mitochondrial Na^+^–Ca^2+^ exchanger, NCLX, in lymphocytes. Cell Calcium.

[16] Samanta K, Mirams GR, Parekh AB (2018). Sequential forward and reverse transport of the Na^+^ Ca^2+^ exchanger generates Ca^2+^ oscillations within mitochondria. Nat Commun.

[17] Luongo TS, Lambert JP, Gross P, Nwokedi M, Lombardi AA, Shanmughapriya S, Carpenter AC, Kolmetzky D, Gao E, van Berlo JH, Tsai EJ, Molkentin JD, Chen X, Madesh M, Houser SR, Elrod JW (2017). The mitochondrial Na^+^/Ca^2+^ exchanger is essential for Ca^2+^ homeostasis and viability. Nature.

[18] Kostic M, Ludtmann MH, Bading H, Hershfinkel M, Steer E, Chu CT, Abramov AY, Sekler I (2015). PKA phosphorylation of NCLX reverses mitochondrial calcium overload and depolarization, promoting survival of PINK1-deficient dopaminergic neurons. Cell Rep.

[19] Jadiya P, Kolmetzky DW, Tomar D, Di Meco A, Lombardi AA, Lambert JP, Luongo TS, Ludtmann MH, Pratico D, Elrod JW (2019). Impaired mitochondrial calcium efflux contributes to disease progression in models of Alzheimer’s disease. Nat Commun.

[20] Crompton M, Capano M, Carafoli E (1976). The sodium-induced efflux of calcium from heart mitochondria. A possible mechanism for the regulation of mitochondrial calcium. Eur J Biochem.

[21] Crompton M, Kunzi M, Carafoli E (1977). The calcium-induced and sodium-induced effluxes of calcium from heart mitochondria. Evidence for a sodium–calcium carrier. Eur J Biochem.

[22] Jung DW, Baysal K, Brierley GP (1995). The sodium–calcium antiport of heart mitochondria is not electroneutral. J Biol Chem.

[23] Affolter H, Carafoli E (1980). The Ca^2+^–Na^+^ antiporter of heart mitochondria operates electroneutrally. Biochem Biophys Res Commun.

[24] Brand MD (1985). The stoichiometry of the exchange catalysed by the mitochondrial calcium/sodium antiporter. Biochem J.

[25] Wingrove DE, Gunter TE (1986). Kinetics of mitochondrial calcium transport. II. A kinetic description of the sodium-dependent calcium efflux mechanism of liver mitochondria and inhibition by ruthenium red and by tetraphenylphosphonium. J Biol Chem.

[26] Kim B, Matsuoka S (2008). Cytoplasmic Na^+^-dependent modulation of mitochondrial Ca^2+^ via electrogenic mitochondrial Na^+^–Ca^2+^ exchange. J Physiol.

[27] Dash RK, Beard DA (2008). Analysis of cardiac mitochondrial Na^+^–Ca^2+^ exchanger kinetics with a biophysical model of mitochondrial Ca^2+^ handling suggests a 3:1 stoichiometry. J Physiol.

[28] Matlib MA, Zhou Z, Knight S, Ahmed S, Choi KM, Krause-Bauer J, Phillips R, Altschuld R, Katsube Y, Sperelakis N, Bers DM (1998). Oxygen-bridged dinuclear ruthenium amine complex specifically inhibits Ca^2+^ uptake into mitochondria in vitro and in situ in single cardiac myocytes. J Biol Chem.

[29] Cox DA, Conforti L, Sperelakis N, Matlib MA (1993). Selectivity of inhibition of Na^+^–Ca^2+^ exchange of heart mitochondria by benzothiazepine CGP-37157. J Cardiovasc Pharmacol.

[30] Griffiths EJ (1999). Reversal of mitochondrial Na/Ca exchange during metabolic inhibition in rat cardiomyocytes. FEBS Lett.

[31] Smets I, Caplanusi A, Despa S, Molnar Z, Radu M, VandeVen M, Ameloot M, Steels P (2004). Ca^2+^ uptake in mitochondria occurs via the reverse action of the Na^+^/Ca^2+^ exchanger in metabolically inhibited MDCK cells. Am J Physiol Renal Physiol.

[32] Murphy E, Eisner DA (2009). Regulation of intracellular and mitochondrial sodium in health and disease. Circ Res.

[33] Despa S, Bers DM (2013). Na^+^ transport in the normal and failing heart—remember the balance. J Mol Cell Cardiol.

[34] Santulli G, Xie WJ, Reiken SR, Marks AR (2015). Mitochondrial calcium overload is a key determinant in heart failure. Proc Natl Acad Sci USA.

[35] Liu T, O’Rourke B (2008). Enhancing mitochondrial Ca^2+^ uptake in myocytes from failing hearts restores energy supply and demand matching. Circ Res.

[36] Hilgemann DW, Matsuoka S, Nagel GA, Collins A (1992). Steady-state and dynamic properties of cardiac sodium–calcium exchange. Sodium-dependent inactivation. J Gen Physiol.

[37] Czyz A, Kiedrowski L (2003). Inhibition of plasmalemmal Na^+^/Ca^2+^ exchange by mitochondrial Na^+^/Ca^2+^ exchange inhibitor 7-chloro-5-(2-chlorophenyl)-1,5-dihydro-4,1-benzothiazepin-2(3H)-one (CGP-37157) in cerebellar granule cells. Biochem Pharmacol.

[38] Neumann JT, Diaz-Sylvester PL, Fleischer S, Copello JA (2011). CGP-37157 inhibits the sarcoplasmic reticulum Ca^2+^ ATPase and activates ryanodine receptor channels in striated muscle. Mol Pharmacol.

[39] Drumm BT, Sung TS, Zheng H, Baker SA, Koh SD, Sanders KM (2018). The effects of mitochondrial inhibitors on Ca^2+^ signalling and electrical conductances required for pacemaking in interstitial cells of Cajal in the mouse small intestine. Cell Calcium.

